# UK clinical guideline for the prevention and treatment of osteoporosis

**DOI:** 10.1007/s11657-017-0324-5

**Published:** 2017-04-19

**Authors:** J. Compston, A. Cooper, C. Cooper, N. Gittoes, C. Gregson, N. Harvey, S. Hope, J. A. Kanis, E. V. McCloskey, K. E. S. Poole, D. M. Reid, P. Selby, F. Thompson, A. Thurston, N. Vine, The National Osteoporosis Guideline Group (NOGG)

**Affiliations:** 1 Department of Medicine, Cambridge Biomedical Campus, Cambridge, UK; 2 Crawley Fracture Liaison Service, Crawley, Sussex, UK; 3 MRC Lifecourse Epidemiology Unit, University of Southampton, Southampton, UK; 4 University Hospitals Birmingham NHS Foundation Trust, Centre for Endocrinology, Diabetes and Metabolism, University of Birmingham & Birmingham Health Partners, Birmingham, UK; 5 Musculoskeletal Research Unit, University of Bristol and Royal United Hospital NHS Foundation Trust, Bath, UK; 6 Metabolic Bone, Nuffield Orthopaedic Hospital, Oxford, UK; 7 Centre for Metabolic Diseases, University of Sheffield Medical School, Sheffield, UK; 8 Metabolic Bone, University of Sheffield, Sheffield, UK; 9 Emeritus Professor of Rheumatology, University of Aberdeen, Aberdeen, UK; 10 Metabolic Bone Disease, University of Manchester, Manchester, UK; 11 National Osteoporosis Society, Camerton, UK

**Keywords:** Osteoporosis, Fracture, NOGG, Guideline

## Abstract

**Introduction:**

In 2008, the UK National Osteoporosis Guideline Group (NOGG) produced a guideline on the prevention and treatment of osteoporosis, with an update in 2013. This paper presents a major update of the guideline, the scope of which is to review the assessment and management of osteoporosis and the prevention of fragility fractures in postmenopausal women and men age 50 years or over.

**Methods:**

Where available, systematic reviews, meta-analyses and randomised controlled trials were used to provide the evidence base. Conclusions and recommendations were systematically graded according to the strength of the available evidence.

**Results:**

Review of the evidence and recommendations are provided for the diagnosis of osteoporosis, fracture-risk assessment, lifestyle measures and pharmacological interventions, duration and monitoring of bisphosphonate therapy, glucocorticoid-induced osteoporosis, osteoporosis in men, postfracture care and intervention thresholds.

**Conclusion:**

The guideline, which has received accreditation from the National Institute of Health and Care Excellence (NICE), provides a comprehensive overview of the assessment and management of osteoporosis for all healthcare professionals who are involved in its management.

## Introduction

This updated guideline provides guidance on the prevention and treatment of osteoporosis in the UK. It updates guidelines previously developed by the Royal College of Physicians [1, 2] and the National Osteoporosis Guideline Group [3, 4]. The scope of the guideline is to review the assessment and diagnosis of osteoporosis, the therapeutic interventions available and the manner in which these can be used to develop management strategies for the prevention of fragility fracture in postmenopausal women and in men age 50 years or over. The guideline was prepared by a writing group (Appendix 1) and finalised after consultation with stakeholders (Appendix 2).

The guideline is intended for all healthcare professionals involved in the management of osteoporosis. The conclusions and recommendations in the document are systematically graded, according to the quality of information available, to indicate the level of evidence on which recommendations are based. The grading methodology is summarised in Appendix 3. Where available, systematic reviews, meta-analyses and randomised controlled trials have been used to provide the evidence base. The evidence base was updated using PubMed to identify systematic reviews and meta-analyses from January 2009 to June 2016. The recommendations in this guideline were agreed unanimously by the National Osteoporosis Guideline Development Group.

The recommendations in the guideline should be used to aid management decisions but do not replace the need for clinical judgement in the care of individual patients in clinical practice.

## Background

Osteoporosis is described by the World Health Organisation (WHO) as a ‘progressive systemic skeletal disease characterized by low bone mass and microarchitectural deterioration of bone tissue, with a consequent increase in bone fragility and susceptibility to fracture’ [5]. The clinical significance of osteoporosis lies in the fractures that arise. In the UK, approximately 536,000 new fragility fractures occur each year, comprising 79,000 hip fractures, 66,000 clinically diagnosed vertebral fractures, 69,000 forearm fractures and 322,000 other fractures (i.e. fractures of the pelvis, rib, humerus, tibia, fibula, clavicle, scapula, sternum and other femoral fractures) [6]. Such fractures cause severe pain and disability to individual sufferers, at an annual cost to the National Health Service (NHS) of over £4.4 billion, estimated for 2010. First year costs, subsequent year costs and pharmacological fracture prevention costs amounted to £3.2 billion, £1.1 billion and £84 million, respectively [6]. More than one third of adult women and one in five men will sustain one or more fragility fractures in their lifetime [7].

Common sites of fragility fracture include the vertebral bodies, distal radius, proximal humerus, pelvis and proximal femur. Hip fractures account for occupation of over 4000 beds at any one time across England, Wales and Northern Ireland and an average hospital length of stay of around 20 days [8]. Hip fractures account for around 50% of the total cost of fractures to the UK annually [6]. Approximately 53% of patients suffering a hip fracture can no longer live independently and 28.7% die within 12 months of the fracture. Only 54% of individuals admitted from home with a hip fracture return there within 30 days [8, 9]. Furthermore, most major fractures are associated with reduced relative survival, with an impact persisting more than 5 years after the index event [10, 11].

In the UK, fracture rates vary by geographic location, socioeconomic status and ethnicity [12, 13] and changes in age- and sex-adjusted fracture rates have been observed in recent decades, with increases in hip fractures amongst men and vertebral fracture amongst women [14]. Furthermore, the ageing of the UK population will give rise to a doubling in the number of fragility fractures over the next 50 years if changes are not made to current practice [6, 15]. Fall-related risk factors add significantly to the risk of fracture and often overlap with risk factors for osteoporosis. Identification of older people at risk of fracture should therefore involve an integrated approach [16].

## Definition and diagnosis of osteoporosis

Prospective studies have shown that the risk of fracture increases progressively with decreasing bone mineral density (BMD). Systematic review and meta-analysis of observational population-based studies using absorptiometric techniques indicate that the risk of fracture increases approximately 2-fold for each standard deviation (SD) decrease in BMD [17, 18] (Evidence level Ia). The predictive value of BMD for hip fracture is at least as good as that of blood pressure for stroke.

Osteoporosis is defined operationally on the level of bone mass, measured as BMD. Two thresholds of BMD have been defined by the World Health Organisation, on the basis of the relationship of fracture risk to BMD. ‘Osteoporosis’ denotes a value for BMD that is 2.5 SDs or more below the young adult mean value for women (T-score equal to or less than −2.5). ‘Severe’ or ‘established’ osteoporosis denotes osteoporosis as defined above in the presence of one or more documented fragility fractures [5].

The World Health Organisation and the International Osteoporosis Foundation recommend that the reference technology for the diagnosis of osteoporosis is dual-energy X-ray absorptiometry (DXA) applied to the femoral neck. The femoral neck is the preferred site because of its higher predictive value for fracture risk [19, 20] (Evidence level 1a). The spine is not a suitable site for diagnosis in older people because of the high prevalence of degenerative changes, which artefactually increase the BMD value; however, it is the preferred site for assessing response to treatment [21]. The normal reference range in men and women is that derived from the NHANES survey for Caucasian women age 20–29 years [20]. The writing group endorses these recommendations (Grade C recommendation). Other sites and validated technologies may be used in clinical practice, but it should be recognised that the significance of a given T-score differs between sites and technologies [22] (Grade B recommendation).

Femoral neck and total hip T-scores calculated from two-dimensional projections of quantitative computed tomography (QCT) data are equivalent to the corresponding DXA-derived T-scores used for the diagnosis of osteoporosis [21, 23].

On GE Healthcare bone densitometers, there is an option for T-scores for men to be given relative to either the male or female reference range in DXA readouts. The same diagnostic cutoff values for BMD can be applied to men as for women since there is evidence that the risk of fracture for any given femoral neck BMD and age is similar in men to that in women [24, 25] (Grade B recommendation).

Some guidelines favour the concurrent use of BMD at the proximal femur and at the lumbar spine for patient assessment. Patients are defined as having osteoporosis on the basis of the lower of the two T-scores. The prediction of fracture is, however, not improved by the use of multiple sites [26, 27] (Evidence level II) and the use of multiple sites for diagnosis is not recommended (Grade B recommendation). However, where hip measurement is not possible for technical reasons or in younger postmenopausal women and men in whom the spine is differentially affected, spine BMD measurements may be used. If neither hip nor spine measurements are possible, BMD measurements at the distal radius may be considered.

Additional techniques for assessing skeletal status have been less well validated than absorptiometric techniques. The writing group does not recommend the use of other techniques, including quantitative ultrasound, for the diagnosis of osteoporosis. This does not preclude the use of these or other validated techniques in risk assessment.

## Fracture-risk assessment

In addition to its diagnostic use, the assessment of BMD provides information on the likelihood of future fractures. The risk of fracture increases approximately 2-fold for each SD decrease in BMD, but the gradient of risk (RR/SD) varies according to the site and technique used, the patient’s age and the fracture outcome [18] (Evidence level Ia).

The use of BMD alone to assess fracture risk has a high specificity but low sensitivity. The low sensitivity over most assumptions means that most fragility fractures will occur in women who do not have osteoporosis as defined by a T-score ≤ −2.5 [28] (Evidence level Ia). The working group does not recommend the use of BMD testing alone for population screening [29] (Grade B recommendation).

Techniques of clinical value include DXA at the hip regions, lumbar spine and forearm. DXA measurements of femoral neck BMD are used in FRAX. Other non-invasive techniques include quantitative ultrasound and computed axial tomography. No one technique subserves all the functions of skeletal assessment (diagnosis, prognosis and monitoring of treatment).

The performance characteristics of BMD assessment can be improved by the concurrent consideration of risk factors that operate independently of BMD. Of particular importance is age, which contributes to risk independently of BMD [30, 31] (Evidence level Ia).

Several additional clinical risk factors have been identified that provide information on fracture risk independently of both age and BMD (Evidence level Ia).

- Low body mass index (BMI). Low BMI is a significant risk factor for hip fracture, but the value of BMI in predicting other fractures is very much diminished when adjusted for BMD [32] (Evidence level 1a).
- A history of a prior fracture at a site characteristic for osteoporosis is an important risk factor for further fracture. Fracture risk is approximately doubled in the presence of a prior fracture, including morphometric vertebral fractures. The increase in risk is even more marked for more than one vertebral fracture. The risks are in part independent of BMD [33] (Evidence level 1a).
- A parental history of hip fracture is a significant risk factor that is largely independent of BMD [34] (Evidence level 1a).
- Smoking is a risk factor that is in part dependent on BMD [35] (Evidence level 1a).
- Glucocorticoids increase fracture risk in a dose-dependent manner. The fracture risk conferred by the use of glucocorticoids is, however, not solely dependent upon bone loss and BMD-independent risks have been identified [36, 37] (Evidence level 1a).
- Alcohol. The relationship between alcohol intake and fracture risk is dose-dependent. Where alcohol intake is on average 2 units or less daily, no increase in risk has been identified. Intakes of 3 or more units daily are associated with a dose-dependent increase in fracture risk [38] (Evidence level 1a).
- Rheumatoid arthritis. There are many secondary causes of osteoporosis (e.g. inflammatory bowel disease, endocrine disorders), but in most instances, it is uncertain to what extent this is dependent on low BMD or other factors such as the use of glucocorticoids. By contrast, rheumatoid arthritis increases fracture risk independently of BMD and the use of glucocorticoids [37] (Evidence level 1a). Recent information suggests that diabetes (particularly type 2) may also exert BMD-independent effects on fracture risk [39, 40].

The consideration of these risk factors improves the sensitivity of testing without sacrificing specificity, and the writing group recommend their inclusion in case-finding algorithms (Grade B recommendation). Indeed, the use of combined clinical risk factors alone performs very similarly to that of BMD alone [41]; the use of clinical risk factors with the addition of BMD is optimal, but the latter can be included in targeted groups (see below).

There are many additional risk factors for fracture that act solely by reducing BMD and others that have been less well validated or identify a risk that may not be amenable to particular treatments. Liability to falls is an appropriate example where the risk of fracture is high, but treatment with agents affecting bone metabolism have an uncertain effect on fracture risk in such patients. The writing group recommend the identification and validation of additional clinical risk factors as an important area for further research.

Biochemical indices of skeletal turnover have the potential to aid risk assessment but probably play a more immediate role in the monitoring of treatment [42] (Evidence level Ia). Further research in this field is recommended so that their utility in clinical practice can be evaluated for use in diagnosis, prognosis and monitoring of treatment [43].

The International Osteoporosis Foundation recommends that risk of fracture should be expressed as an absolute risk, i.e. probability over a 10-year interval. The absolute risk of fracture depends upon age and life expectancy as well as the current relative risk. The period of 10 years covers the likely initial duration of treatment and the benefits that may continue if treatment is stopped. The writing group endorses these recommendations (Grade C recommendation).

Algorithms that integrate the weight of clinical risk factors for fracture risk, with or without information on BMD, have been developed by the WHO Collaborating Centre for Metabolic Bone Diseases at Sheffield. The FRAX tool (www.shef.ac.uk/FRAX) computes the 10-year probability of hip fracture or a major osteoporotic fracture. A major osteoporotic fracture is a clinical spine, hip, forearm or humerus fracture. The tool has been externally validated in independent cohorts [30] (Evidence level Ia). QFracture is based on a UK prospective open cohort study of routinely collected data from general practises that takes into account numerous risk factors and estimates the 1–10-year cumulative incidence of hip or major osteoporotic fracture [44]; [http://www.qfracture.org]. The National Institute for Health and Care Excellence (NICE) has recommended the use of fracture risk assessment tools (FRAX or QFracture) in the assessment of patients, including the proposal that their use should be considered in all women age 65 years or older and men age 75 years or older [29]. In the Scottish Intercollegiate Guidelines Network guideline (SIGN 142), QFracture is preferred and is used to provide a threshold for BMD assessment [45]. Since FRAX and QFracture yield different outputs (probability of fracture accounting for mortality risk in the case of FRAX, and a cumulative risk of fracture in the case of QFracture), the two calculators cannot be used interchangeably. In addition, BMD cannot be incorporated into QFracture estimations. Finally, the National Osteoporosis Guideline Group (NOGG) intervention thresholds are based on FRAX probability and thus cannot be used with fracture risk derived from QFracture or other calculators [46]. The use of FRAX for fracture risk assessment is therefore preferred (Grade B recommendation).

The FRAX assessment takes no account of prior treatment or of dose responses for several risk factors. For example, two prior fractures carry a much higher risk than a single prior fracture. Dose responses are also evident for glucocorticoid use and are partially addressed in the NOGG guideline. A prior clinical vertebral fracture carries an approximately 2-fold higher risk than other prior fractures. Since it is not possible to model all such scenarios with the FRAX algorithm, these limitations should temper clinical judgement.

Diagnostic assessment of individuals with osteoporosis should include not only the assessment of BMD where indicated but also the exclusion of diseases that mimic osteoporosis, elucidation of the cause of the osteoporosis and the management of any associated morbidity. Recommendations for the routine investigation of patients with osteoporosis are shown in Table 1.

**Table 1:** Procedures proposed in the investigation of osteoporosis

Routine
• History and physical examination
• Blood cell count, sedimentation rate or C-reactive protein. Serum calcium, albumin, creatinine, phosphate, alkaline phosphatase and liver transaminases
• Thyroid function tests
• Bone densitometry (DXA)
Other procedures, if indicated
• Lateral radiographs of lumbar and thoracic spine or DXA-based lateral vertebral imaging
• Serum protein immunoelectrophoresis and urinary Bence Jones proteins
• Serum 25-hydroxyvitamin D
• Plasma parathyroid hormone
• Serum testosterone, sex hormone binding globulin, follicle stimulating hormone, luteinizing hormone (in men)
• Serum prolactin
• 24 h urinary free cortisol/overnight dexamethasone suppression test
• Endomysial and/or tissue transglutaminase antibodies
• Isotope bone scan
• Markers of bone turnover
• Urinary calcium excretion

Other investigations, for example, bone biopsy and genetic testing for osteogenesis imperfecta, are largely restricted to specialist centres

The majority of vertebral fractures do not come to medical attention and thus remain undiagnosed [47]. Moderate or severe vertebral fractures, even when asymptomatic, are strong risk factors for subsequent fracture at the spine and other skeletal sites [48–50]. Vertebral fracture assessment should therefore be considered in high-risk individuals, using either lateral lumbar and thoracic spine radiographs or lateral spine DXA imaging. The latter delivers a significantly lower radiation dose but performs comparably to traditional radiographs [51].

Vertebral fracture assessment should be considered in postmenopausal women and older men if there is a history of ≥4 cm height loss, kyphosis, recent or current long-term oral glucocorticoid therapy, or a BMD T-score ≤ −2.5 (Grade C recommendation). It should also be considered in individuals with a history of non-vertebral fracture after the age of 50 years [52].

## Lifestyle measures in the management of osteoporosis

Lifestyle measures to improve bone health include increasing the level of physical activity, stopping smoking, reducing alcohol intake to ≤2 units/day, reducing the risk of falls and ensuring adequate dietary calcium intake and vitamin D status.

Increasing calcium intake, either through the diet or in the form of supplements, has been shown to result in small increases in BMD [53] (Evidence level 1a) but convincing evidence that calcium alone reduces fracture risk is lacking [54, 55] (Evidence level 1a). Calcium supplements are associated with an increased risk of nephrolithiasis [56] and gastrointestinal side-effects. Concerns have also been raised that calcium supplements increase the risk of cardiovascular disease, but in a recent meta-analysis little evidence was found for a significant association [57] (Evidence level 1a). It is recommended that a daily calcium intake of between 700 and 1200 mg should be advised, if possible achieved through dietary intake (https://www.gov.uk/government/uploads/…/familyfood-method-rni-11dec14.pdf) (Grade B recommendation). A simple dietary calcium intake calculator is available at http://www.cgem.ed.ac.uk/research/rheumatological/calcium-calculator.

The Scientific Advisory Committee on Nutrition (SACN) has recently recommended a reference nutrient intake (RNI) of 400 IU daily for adults of all ages [58]. However, in postmenopausal women and older men at increased risk of fracture, the available evidence supports the use of higher doses. Vitamin D alone is ineffective in reducing fracture risk but when combined with calcium supplements results in a small reduction in hip and non-vertebral fractures, and possibly also in vertebral fractures [59, 60] (Evidence level 1a). In another meta-analysis, a protective effect of vitamin D on fractures was only seen at daily doses ≥800 IU (20 μg) [61] (Evidence level 1a). This dose of vitamin D may also reduce falls [62] (Evidence level 1a). It is recommended that in postmenopausal women and men ≥50 years who are at increased risk of fracture, a daily dose of 800 IU of cholecalciferol should be advised (Grade A recommendation). Intermittent administration of large doses of vitamin D, e.g. ≥100,000 IU is not advised, based on recent reports of an associated increased risk of fracture and falls [63, 64].

Supplementation with calcium and vitamin D is often advocated as an adjunct to other treatments for osteoporosis, as the clinical trials of these agents were performed in patients who were calcium and vitamin D replete. In postmenopausal women and older men receiving bone-protective therapy for osteoporosis it is recommended that calcium supplementation should also be given if the dietary intake is below 700 mg/day, and vitamin D supplementation with 800 IU/day of cholecalciferol considered in those at risk of/with evidence for vitamin D insufficiency (Grade B recommendation).

Weight-bearing exercise has beneficial effects on BMD [65] (Evidence level 1a) but has not been shown to reduce fracture risk [66] (Evidence level 1a). Regular weight-bearing exercise should be advised, tailored according to the individual patient (Grade B recommendation). Physiotherapy is an important component of rehabilitation after fracture. Muscle strengthening and balance training exercise interventions may reduce falls by improving confidence and coordination as well as maintaining bone mass.

The majority of fractures are preceded by a fall. Multi-component group and home-based exercise programmes, Tai Chi and home safety interventions have been shown to reduce the risk of falls in people living in the community [67] (Evidence level 1a). Falls prevention exercise programmes in community dwelling adults age >60 years may reduce falls resulting in fracture [68] (Evidence level 1a) although in individuals at higher risk of falling, this benefit has not been shown. Falls history should be obtained in patients with osteoporosis and further assessment and appropriate measures undertaken in those at risk (Grade B recommendation).

Hip protectors may reduce the risk of hip fractures in older people in nursing care or residential care settings [69] (Evidence level 1a). However, poor acceptance and adherence by older people offered hip protectors are barriers to their use.

Sufficient protein intake is necessary to maintain the function of the musculoskeletal system and also decreases the complications that occur after hip fracture. Protein supplementation in patients with a recent hip fracture has been shown to improve the subsequent clinical course by significantly lowering the rate of infection and duration of hospital stay [70] (Evidence level Ib).

## Pharmacological interventions

In the context of strategies for treating individuals at high risk of fracture, no distinction is made between prevention and treatment. A range of pharmacological interventions has been shown to be effective in reducing fracture risk in postmenopausal women with osteoporosis [71]. Recommendations concerning the major interventions for osteoporosis are based on high levels of evidence (Evidence level 1a and Ib), and the grade of these recommendations is summarised in Table 2.

**Table 2:** Anti-fracture efficacy of approved treatments for postmenopausal women with osteoporosis when given with calcium and vitamin D

Intervention	Vertebral fracture	Non-vertebral fracture	Hip fracture
Alendronate	A	A	A
Ibandronate	A	A*	NAE
Risedronate	A	A	A
Zoledronic acid	A	A	A
Calcitriol	A	NAE	NAE
Denosumab	A	A	A
HRT	A	A	A
Raloxifene	A	NAE	NAE
Teriparatide	A	A	NAE

*A* grade A recommendation, *NAE* not adequately evaluated, HRT hormone replacement therapy

*In subsets of patients only (post hoc analysis)

Bisphosphonates are analogues of inorganic pyrophosphate that inhibit bone resorption.

- *Alendronate* is approved for the treatment of postmenopausal osteoporosis (10 mg daily or 70 mg once weekly by mouth) and osteoporosis in men (10 mg daily). It is also approved for the prevention of postmenopausal osteoporosis (5 mg daily) and for prevention and treatment of glucocorticoid-induced osteoporosis (5 mg daily or, in postmenopausal women not receiving hormone replacement therapy 10 mg daily).

In postmenopausal women with osteoporosis, alendronate at 10 mg daily has been shown to reduce vertebral, non-vertebral and hip fractures [72]. Approval for the use of alendronate in men with osteoporosis and in men and women taking glucocorticoids was granted on the basis of BMD bridging studies [73, 74]. Side-effects include upper gastrointestinal symptoms, bowel disturbance, headaches and musculoskeletal pain.

Alendronate should be taken after an overnight fast and at least 30 min before the first food or drink (other than water) of the day or any other oral medicinal products or supplementation (including calcium). Tablets should be swallowed whole with a glass of plain water (∼200 ml) while the patient is sitting or standing in an upright position. Patients should not lie down for 30 min after taking the tablet. Alendronic acid is also available as 70 mg effervescent or soluble tablets, to be dissolved in half a glass of plain water (≥120 ml).

b) *Ibandronate at* 150 mg once monthly by mouth or 3 mg as an intravenous injection every 3 months is approved for the treatment of osteoporosis in postmenopausal women at increased risk of fracture.

In a dose of 2.5 mg daily by mouth, a significant reduction in vertebral fractures was demonstrated [75]. In a post hoc analysis of high-risk women (femoral neck BMD T-score below −3.0), a significant reduction in non-vertebral fractures was shown [76]. No data are available for hip fracture. Approval for the oral 150 mg once monthly and 3 mg intravenously every 3-month formulations was granted on the basis of BMD bridging studies.

Side-effects with the oral preparation include upper gastrointestinal side-effects and bowel disturbance. Intravenous administration may be associated with an acute phase reaction, characterised by an influenza-like illness; this is generally short-lived and typically occurs only after the first injection.

Oral ibandronate should be taken after an overnight fast and 1 h before the first food or drink (other than water) of the day or any other oral medicinal products or supplementation (including calcium). Tablets should be swallowed whole with a glass of plain water (180 to 240 ml) while the patient is sitting or standing in an upright position. Patients should not lie down for 1 h after taking the tablet.

c) *Risedronate* at 5 mg daily or 35 mg once weekly by mouth is approved for the treatment of postmenopausal osteoporosis, to reduce the risk of vertebral fracture and for the treatment of established postmenopausal osteoporosis, to reduce the risk of hip fractures. It is also indicated for the treatment of osteoporosis in men at high risk of fractures. Risedronate at 5 mg daily is approved for the prevention of glucocorticoid-induced osteoporosis in postmenopausal women.

In postmenopausal women with osteoporosis, risedronate at 5 mg daily has been shown to reduce vertebral and non-vertebral fractures [77, 78]. In a large population of older women, risedronate significantly decreased the risk of hip fractures, an effect that was greater in osteoporotic women [79]. Approval for use of risedronate in men with osteoporosis and in postmenopausal women taking glucocorticoids was granted on the basis of BMD bridging studies [80–82]. Side-effects include upper gastrointestinal symptoms, bowel disturbance, headache and musculoskeletal pain.

Risedronate should be taken after an overnight fast and at least 30 min before the first food or drink (other than water) of the day or any other oral medicinal products or supplementation (including calcium). Tablets should be swallowed whole with a glass of plain water (∼120 ml) while the patient is sitting or standing in an upright position. Patients should not lie down for 30 min after taking the tablet.

d) *Zoledronic acid* at 5 mg intravenously once yearly is approved for the treatment of osteoporosis in postmenopausal women and men at increased risk of fracture, including those with a recent low trauma fracture, and for the treatment of osteoporosis associated with long-term systemic glucocorticoid therapy in postmenopausal women and men.

Zoledronic acid has been shown to reduce the incidence of vertebral, non-vertebral and hip fractures in postmenopausal women with osteoporosis [83] and to reduce the risk of clinical fracture and attendant mortality when given to patients shortly after their first hip fracture [84]. Approval for its use in men with osteoporosis and postmenopausal women and men taking glucocorticoids was granted on the basis of BMD bridging studies [85, 86]. Side-effects include an acute phase reaction (see above), usually only after the first infusion, and gastrointestinal symptoms. Creatinine clearance should be calculated (e.g. using the Cockroft-Gault formula (140 − age (years) × weight (kg) × *f*/serum creatinine (μmol/l) where *f* = 1.23 for men and 1.04 for women) prior to initiation of treatment and serum creatinine monitored in high-risk patients. An increase in atrial fibrillation, reported as a serious adverse event, was seen in the main phase III trial although this finding has not been replicated in other trials involving zoledronic acid. Zoledronic acid is given as an intravenous infusion over a minimum period of 15 min.

e) Contraindications and special precautions for the use of bisphosphonates

Oral and intravenous bisphosphonates are contraindicated in patients with hypocalcaemia, hypersensitivity to bisphosphonates, and severe renal impairment (GFR ≤35 ml/min for alendronate and zoledronic acid and ≤30 ml/min for other bisphosphonates). Pregnancy and lactation are also contraindications. Oral bisphosphonates are contraindicated in people with abnormalities of the oesophagus that delay oesophageal emptying such as stricture or achalasia, and inability to stand or sit upright for at least 30–60 min. They should be used with caution in patients with other upper gastrointestinal disorders. Pre-existing hypocalcaemia must be investigated and, where due to vitamin D deficiency, treated with vitamin D (e.g. 50,000 to 100,000 IU orally as a loading dose) before treatment is initiated.

Rare adverse effects, in particular, osteonecrosis of the jaw and atypical femoral fractures, have led to additional precautions. In patients with dental disease or other risk factors (e.g. glucocorticoids, tobacco use), dental examination with preventive dentistry is recommended prior to treatment with oral or intravenous bisphosphonates. While on treatment, patients should avoid invasive dental procedures if possible. For patients requiring dental procedures, there are no data available to indicate whether discontinuation of treatment reduces the risk of osteonecrosis of the jaw. Clinical judgement of the treating physician should guide the management plan of each patient based on individual benefit/risk assessment. During treatment, all patients should be encouraged to maintain good oral hygiene, receive routine dental check-ups, and report any oral symptoms such as dental mobility, pain or swelling.

The possibility of osteonecrosis of the external auditory canal should be considered in patients who present with ear symptoms including chronic ear infections. Possible risk factors for osteonecrosis of the external auditory canal include steroid use and chemotherapy and/or local risk factors such as infection or trauma.

During treatment, patients should be advised to report any thigh, hip or groin pain and any patient presenting with such symptoms should be evaluated for possible atypical femur fracture.

Denosumab is a fully humanised monoclonal antibody against Receptor Activator of Nuclear factor Kappa B Ligand (RANKL), a major regulator of osteoclast development and activity. It is approved for the treatment of osteoporosis in postmenopausal women and men at increased risk of fractures, and for the treatment of bone loss associated with hormone ablation in men with prostate cancer at increased risk of fractures. It is given as a subcutaneous injection of 60 mg once every 6 months.

Denosumab has been shown to reduce the incidence of vertebral, non-vertebral and hip fractures in postmenopausal women with osteoporosis [87]. Approval for its use in men with osteoporosis was granted on the basis of a BMD bridging study [88].

### Contraindications and special precautions

Denosumab is contraindicated in women with hypocalcaemia or with hypersensitivity to any of the constituents of the formulation. Its use is not recommended in pregnancy or in the paediatric population (age ≤18 years). Side-effects include skin infection, predominantly cellulitis, and hypocalcaemia.

Hypocalcaemia is an identified risk in patients treated with denosumab, which increases with the degree of renal impairment. Pre-existing hypocalcaemia must be investigated and, where due to vitamin D deficiency, treated with vitamin D (e.g. 50,000 to 100,000 IU orally as a loading dose) before treatment is initiated. Adequate intake of calcium and vitamin D is important in all patients, especially in those with severe renal impairment.

Monitoring of calcium levels should be conducted prior to each dose of denosumab and within 2 weeks after the initial dose in patients predisposed to hypocalcaemia (e.g. patients with severe renal impairment, creatinine clearance ≤30 ml/min) or if suspected symptoms of hypocalcaemia occur or if otherwise indicated. Patients should be advised to report symptoms of hypocalcaemia.

The rare occurrence of osteonecrosis of the jaw and atypical femoral fractures in patients treated with denosumab has led to additional precautions. In patients with dental disease or other risk factors (e.g. glucocorticoid therapy, tobacco use), dental examination with preventive dentistry is recommended prior to treatment. While on treatment, patients should avoid invasive dental procedures if possible. For patients requiring dental procedures, there are no data available to indicate whether discontinuation of treatment reduces the risk of osteonecrosis of the jaw. Clinical judgement of the treating physician should guide the management plan of each patient based on individual benefit/risk assessment. During treatment, all patients should be encouraged to maintain good oral hygiene, receive routine dental check-ups, and report any oral symptoms such as dental mobility, pain or swelling.

During treatment, patients should be advised to report any thigh, hip or groin pain and any patient presenting with such symptoms should be evaluated for an atypical femur fracture.

Following cessation of denosumab therapy, rapid bone loss occurs [89]. Whether this results in an increase in fracture risk is unclear, but there are case reports of vertebral fractures, often multiple, occurring within 18 months after stopping treatment [90–92]. Although further studies are required, in patients who stop denosumab, switching to an alternative therapy such as a bisphosphonate should be considered (Grade C recommendation).

Raloxifene is a selective oestrogen receptor modulator and inhibits bone resorption. It is approved for the treatment and prevention of osteoporosis in postmenopausal women.

Raloxifene has been shown to reduce vertebral fracture risk but reduction in non-vertebral and hip fractures has not been demonstrated [93]. Raloxifene is contraindicated in women with child-bearing potential, a history of venous thromboembolism or unexplained uterine bleeding. Hepatic impairment and severe renal impairment are also contraindications. It should be used with caution in women with a history of stroke or with risk factors for stroke. Side-effects include leg cramps, oedema and vasomotor symptoms. There is a small increase in the risk of venous thromboembolism, mostly within the first few months of treatment and a small increase in the risk of fatal stroke has been reported. In the phase III trials, women treated with raloxifene had a significantly decreased risk of developing breast cancer.

Raloxifene is taken orally as a single daily dose (60 mg) and may be taken at any time without regard to meals.

Teriparatide (recombinant human parathyroid hormone (PTH) 1–34), when administered intermittently, has anabolic skeletal effects which are most marked in cancellous bone. Teriparatide is approved for treatment of osteoporosis in postmenopausal women and in men at high risk of fracture. Teriparatide is also approved for the treatment of osteoporosis associated with systemic glucocorticoid therapy in women and men at increased risk of fracture.

Teriparatide has been shown to reduce vertebral and non-vertebral fractures in postmenopausal women with osteoporosis [97]. No data are available for hip fractures. Approval for its use in men with osteoporosis and in glucocorticoid-induced osteoporosis was granted on the basis of BMD bridging studies [98, 99].

Teriparatide is contraindicated in patients with hypercalcaemia, pregnancy and lactation, metabolic bone diseases other than osteoporosis, severe renal impairment, prior radiation to the skeleton and malignant disease affecting the skeleton. It should be used with caution in patients with moderate renal impairment. Side-effects include headache, nausea, dizziness and postural hypotension. Slight and transient elevations of serum calcium may occur following teriparatide injection.

Teriparatide is given as a subcutaneous injection in a dose of 20 μg/day. The duration of treatment is limited to 24 months.

Calcitriol (1,25-dihydroxyvitamin D) is the active form of vitamin D and is approved for the treatment of established postmenopausal osteoporosis in an oral dose of 0.25 μg twice daily. It acts mainly by inhibiting bone resorption. It has been shown to reduce vertebral fracture risk in postmenopausal women with osteoporosis, but effects on non-vertebral and hip fractures have not been demonstrated [100]. It is contraindicated in patients with hypercalcaemia or with metastatic calcification. Because it may cause hypercalcaemia and/or hypercalciuria, serum calcium and creatinine levels should be monitored at 1, 3 and 6 months after starting treatment and at six monthly intervals thereafter.

Hormone replacement therapy (HRT) comprises a large number of formulations of oestrogen or oestrogen plus progestogen combinations, some of which are approved for the prevention of osteoporosis in postmenopausal women at high risk of fracture. Conjugated equine oestrogens 0.625 mg daily ± 2.5 mg/day of medroxyprogesterone acetate has been shown to reduce vertebral, non-vertebral and hip fractures in postmenopausal women not selected on the basis of low bone density or high fracture risk [101, 102]. Because of the unfavourable risk/benefit balance in older postmenopausal women, the use of HRT for osteoporosis is generally restricted to younger postmenopausal women who are at high risk of fracture and also have menopausal symptoms [103].

No trials have been designed and powered to detect differences in the magnitude of fracture reduction between different treatments. Direct comparison across trials is not possible because of differences in study design, but in general, reductions of 30–70% have been reported for vertebral fracture, up to 20% for non-vertebral fracture and up to 40% for hip fracture.

The choice of agent is determined by the spectrum of anti-fracture effects across skeletal sites, side-effects and cost. The low cost of generic formulations of alendronate and risedronate, which have a broad spectrum of anti-fracture efficacy, make these first line treatments in the majority of cases. In women who are intolerant of oral bisphosphonates or in whom they are contraindicated, intravenous bisphosphonates or denosumab provide appropriate and cost-effective treatment options with hormone replacement therapy or raloxifene as additional options (Grade A recommendation). The high cost of teriparatide restricts its use to those at very high risk, particularly for vertebral fractures.

## Duration and monitoring of bisphosphonate therapy

Concerns over rare adverse effects of long-term bisphosphonate therapy, particularly osteonecrosis of the jaw and atypical femoral fractures, have raised questions about the optimal duration of therapy. Because bisphosphonates are retained in bone for varying periods of time, beneficial effects may persist for some time after cessation of treatment. This has led to the suggestion that some patients may benefit from a period off treatment, in which treatment is stopped after some years and the need for continued therapy is subsequently reassessed. Treatment review in patients taking bisphosphonates is therefore important [104]. Because pivotal clinical trials have mostly been limited to a duration of 3 years, recommendations for longer-term use and for drug holidays are based on limited evidence from extension studies in postmenopausal women [105]. There is currently no evidence on which to base recommendations for men.

Withdrawal of treatment is associated with decreases in BMD and increased bone turnover after 2–3 years for alendronate [106, 107] and 1–2 years for ibandronate and risedronate [108, 109]. In the case of zoledronic acid, withdrawal after 3 years’ treatment was associated with only a very small decrease in BMD after a further 3 years without treatment [110].

In the Fracture Intervention Trial Long-term Extension study of alendronate (FLEX), there were significantly fewer clinical vertebral fractures in women previously treated with alendronate for 5 years who continued with alendronate for five more years than in those assigned to placebo after 5 years of alendronate [107]. In the Health Outcomes and Reduced Incidence with Zoledronic acid Once Yearly (HORIZON) study extension, the risk of morphometric vertebral fractures was significantly lower in women continuing on zoledronic acid for 3 years after 3 years therapy when compared with those switched to placebo, but the risk of non-vertebral fractures was similar in the treatment and placebo groups [110]. Post hoc analyses from the alendronate and zoledronic acid extension studies suggest that women most likely to benefit from long-term bisphosphonate therapy are those with low hip BMD (T-score < −2.0 in FLEX and ≤ −2.5 in HORIZON), those with a prevalent vertebral fracture and those who sustained one or more incident fractures during the initial 3 or 5 years of treatment [111, 112] (Evidence level IIb). Older age was also associated with increased fracture risk after discontinuation of alendronate therapy [113].

Based on the evidence above, continuation of bisphosphonate treatment beyond 3–5 years (3 years for zoledronic acid and 5 years for alendronate, ibandronate and risedronate) can generally be recommended in the following situations: (Evidence level IIb, Grade of recommendation B).

- Age 75 years or more
- Previous history of a hip or vertebral fracture
- Occurrence of one or more low trauma fractures during treatment, after exclusion of poor adherence to treatment (for example less than 80% of treatment has been taken) and after causes of secondary osteoporosis have been excluded
- Current treatment with oral glucocorticoids ≥7.5 mg prednisolone/day or equivalent

If treatment is discontinued, fracture risk should be reassessed:

- After a new fracture regardless of when this occurs
- If no new fracture occurs, after 18 months to 3 years (Grade C recommendation)

**Fig. 1:**
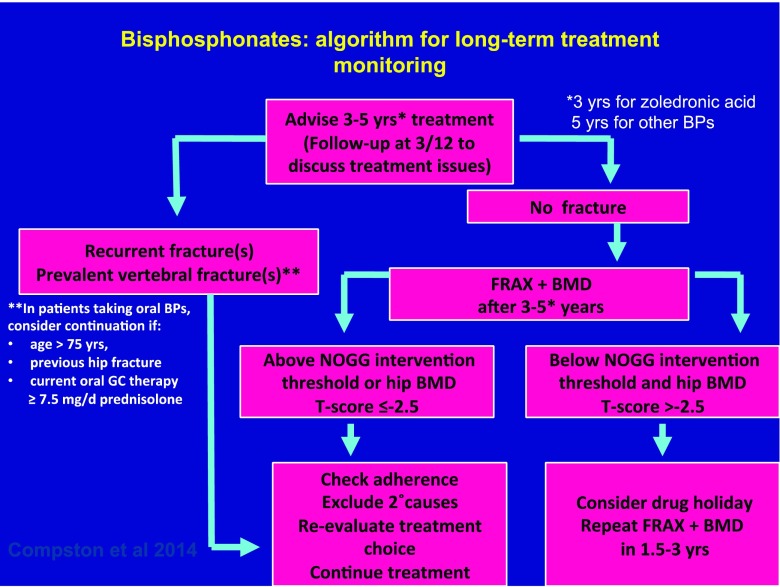
Algorithm for monitoring of long-term bisphosphonate therapy in postmenopausal women

Treatment review should be performed after 5 years of treatment with alendronate, risedronate or ibandronate and after 3 years of treatment with zoledronic acid (Grade C recommendation). Reassessment of fracture risk in treated individuals can be performed using FRAX with femoral neck BMD [114] (Grade B recommendation). The NOGG intervention thresholds can then be used to guide the decision as to whether treatment can be stopped for a period of time (Fig. 1). If the hip BMD T-score is ≤ −2.5, resumption of treatment should be considered regardless of FRAX-derived fracture probability. An algorithm outlining the above approach is shown in Fig. 1.

If biochemical markers of bone turnover indicate relapse from suppressed bone turnover and BMD has decreased following withdrawal, resumption of treatment should be considered (Grade C recommendation).

There is no evidence base to guide decisions about treatment beyond 10 years and management of such patients should be considered on an individual basis.

## Rare long-term adverse effects of bisphosphonates and denosumab

*Osteonecrosis of the jaw* occurs only very rarely in patients receiving bisphosphonate or denosumab therapy for osteoporosis. The estimated incidence in those receiving bisphosphonates is 1–90/100,000 years of patient exposure. Risk factors for osteonecrosis of the jaw include poor oral hygiene, dental disease, dental interventions, cancer, chemotherapy or glucocorticoid therapy [115]. The incidence of osteonecrosis of the jaw is substantially greater with the higher doses of bisphosphonates or denosumab that are used to treat patients with skeletal metastases.

*Atypical femoral fractures*, mainly of the subtrochanteric and diaphyseal regions of the femoral shaft, have been reported rarely in patients taking bisphosphonates or denosumab for osteoporosis. In a recent review by the ASBMR Task Force on the management of osteoporosis in patients on long-term bisphosphonates, a systematic search of the literature revealed wide variation in the relative risk of atypical femoral fractures associated with BP use (between 2- and 128-fold), but the absolute risk was consistently low, ranging between 3.2 and 50 cases/100,000 person-years [116]. This estimate appeared to double with prolonged duration of BP use (> 3 years, median duration 7 years), and declined with discontinuation [116–118].

In a recent nationwide cohort study from Denmark, use of alendronate in excess of 10 years was associated with a 30% lower risk of hip fracture and no increase in the risk of fractures of the subtrochanteric femur and femoral shaft, supporting an acceptable risk benefit balance in terms of fracture outcomes [119].

A typical femoral fractures are often bilateral, associated with prodromal pain and tend to heal poorly. During bisphosphonate or denosumab therapy, patients should be advised to report any unexplained thigh, groin or hip pain and if such symptoms develop, imaging of the femur (X-ray, isotope scanning or MRI) should be performed. If an atypical fracture is present, the contralateral femur should also be imaged. Discontinuation of bisphosphonate or denosumab therapy should be considered in patients who develop an atypical fracture, weight-bearing activity should be restricted and alternative treatment options considered where appropriate. Surgical treatment with intramedullary nailing is often recommended.

## Glucocorticoid-induced osteoporosis

Although guidance on the prevention and management of glucocorticoid osteoporosis has been developed in many countries, there is evidence that osteoporosis risk assessment and management are inadequate in long-term users of oral glucocorticoids [120]. Bone loss and increased fracture risk occur rapidly after initiation of glucocorticoid therapy and increase with the dose and duration of therapy [121, 122]. The increase in fracture risk is seen for vertebral and non-vertebral fractures, including hip fractures, and is partially independent of BMD [37].

Evidence for the efficacy of bone-protective therapy in people receiving glucocorticoids is based mainly on BMD bridging studies [74, 81, 82, 86, 99], although a reduction in vertebral fracture rate has been demonstrated with risedronate in a pooled analysis and with teriparatide in a comparator study (see Table 3) [81, 99, 123].

**Table 3:** Effect of approved interventions for glucocorticoid-induced osteoporosis on BMD and fracture risk

Intervention	Spine BMD	Hip BMD	Vertebral fracture	Non-vertebral fracture
Alendronate	A	A	B^b^	NAE
Risedronate	A	A	A^b^	NAE
Teriparatide	A^a^	A^a^	A^a, b^	NAE
Zoledronic acid	A^a^	A^a^	NAE	NAE

*A* grade A recommendation, *B* grade B recommendation, *NAE* not adequately evaluated

^a^Comparator study

^b^Not a primary endpoint

A working group from the International Osteoporosis Foundation and the European Society of Calcified Tissues published a framework for the development of national guidelines for the management of glucocorticoid-induced osteoporosis in men and women age 18 years or over in whom continuous oral glucocorticoid therapy was considered for 3 months or longer [124, 125]. Evidence for the efficacy of interventions to prevent or treat glucocorticoid-induced osteoporosis was based on an updated systematic literature review from the 2010 American College of Rheumatology Guideline [126]. A summary of the main recommendations is provided below, adapted where appropriate for use in the UK.

FRAX assumes an average dose of prednisolone (2.5–7.5 mg/day or its equivalent) and may underestimate fracture risk in patients taking higher doses and overestimate risk in those taking lower doses. Using UK data, the average adjustments over all ages in postmenopausal women and men age ≥50 years are shown in Table 4 [127].

**Table 4:** Adjustment of FRAX estimates of fracture probability according to dose of prednisolone

Dose	Prednisolone equivalent (mg/day)	Average adjustment: hip fracture probability	Average adjustment: major osteoporotic fracture probability
Low	<2.5	−35%	−20%
Medium	2.5–7.5	None	None
High	≥7.5	+20%	+15%

For high doses of glucocorticoids, for example ≥15 mg prednisolone/day or its equivalent, greater upward adjustment of fracture probability may be required (Grade C recommendation). When the UK FRAX model is used and the glucocorticoid box is filled, 2 points appear on the NOGG graphs, 1 for medium dose and 1 for high dose (all defined as above). The assessment thresholds (fracture probabilities for BMD testing) and intervention thresholds (fracture probabilities for therapeutic intervention) are then used in the same way as described for postmenopausal women and older men (Table 4).

In general, women age ≥70 years, or with a previous fragility fracture or taking large doses of glucocorticoids (≥7.5 mg/prednisolone or equivalent/day) exceed the intervention threshold and should be considered for bone-protective therapy (Grade C recommendation).

Because bone loss and increased fracture risk occur early after initiation of glucocorticoids, bone-protective treatment should be started at the onset of therapy in patients at increased risk of fracture (Grade C recommendation). The low cost of generic formulations of alendronate and risedronate make them first line options in the majority of cases. In individuals who are intolerant of these agents or in whom they are contraindicated, zoledronic acid or teriparatide are appropriate options. Adequate calcium intake should be achieved through dietary intake if possible, with the use of supplements if necessary. An adequate vitamin D status should be maintained, using supplements if required. If glucocorticoid therapy is stopped, withdrawal of bone-protective therapy may be considered, but if glucocorticoids are continued long term, bone protection should be maintained in the majority of cases (Grade C recommendation).

Bone-protective therapy may be appropriate in some premenopausal women and younger men, particularly in individuals with a previous history of fracture or receiving high doses of glucocorticoids (Grade C recommendation). Caution is advised in the use of bisphosphonates in women of child-bearing age. Referral of complex cases to secondary care is recommended (Grade C recommendation).

## Osteoporosis in men

Treatments have been less extensively evaluated in men with osteoporosis than in women, though there is no evidence that skeletal metabolism in men differs fundamentally from that of women [128]. Alendronate, risedronate, zoledronic acid, denosumab and teriparatide are approved for the treatment of osteoporosis in men. Approval has been granted mainly on the basis of BMD bridging studies [73, 80, 85, 88, 98], although reduction in vertebral fractures has also shown in men with osteoporosis treated with alendronate or zoledronic acid [80, 85] (Evidence level 1b).

The low cost of generic formulations of alendronate and risedronate make these first-line treatments in the majority of cases. In men who are intolerant of oral bisphosphonates or in whom they are contraindicated, zoledronic acid or denosumab provide appropriate alternatives, with teriparatide as an additional option (Grade B recommendation).

For the purposes of FRAX calculations, the BMD T-scores in men are calculated based on the female reference database [25] (Grade B recommendation). When FRAX is calculated on densitometers, this is done automatically. When entering data manually to the FRAX calculator, the absolute value of BMD should be used and the manufacturer of the densitometer specified.

Secondary causes of osteoporosis are commonly found amongst men, so this population requires thorough investigation (Grade C recommendation). Intervention thresholds for men are similar to those recommended for women (Grade C recommendation).

All men starting on androgen-deprivation therapy should have their fracture risk assessed (https://www.nice.org.uk/guidance/cg175/chapter/1Recommendations#men-having-hormone-therapy-2) (Grade B recommendation).

Consideration should be given to referring men with osteoporosis to specialist centres, particularly younger men or those with severe disease (Grade C recommendation).

## Postfracture care and fracture liaison services

Collaboration between geriatricians, orthopaedic surgeons and primary care practitioners and between the medical and non-medical disciplines concerned should be encouraged wherever possible. The Department of Health state that Fracture Liaison Services (FLS) should be provided for all patients sustaining a fragility fracture [129].

FLS provide fully coordinated, intensive models of care for secondary fracture prevention. They are cost-effective and are more effective in improving patient outcomes than approaches involving GP and/or patient alerts and/or patient education only. The ideal approach is a service in which identification, assessment and osteoporosis treatment are all conducted within an integrated electronic health care network, overseen by a coordinator and utilising a dedicated database measuring performance [130, 131] (Evidence level 1a).

Coordinator-based FLS systems are recommended, with a dedicated employee (a FLS coordinator) who, using electronic patient lists, systematically identifies men and women with fragility fracture, facilitating clinical risk factor evaluation, pathology tests to exclude secondary causes of osteoporosis and radiological investigation including BMD testing (Grade A recommendation).

The FLS coordinator should either initiate appropriate non-pharmacological and pharmacological interventions or make a treatment recommendation for the primary care physician to initiate. FLS should be provided by a multidisciplinary team, which includes an orthopaedic surgeon, and should be led by a clinician.

FLS should provide a coordinated programme with an integrated approach for falls and fracture prevention. All individuals with fracture should be fully assessed for falls risk factors and appropriate interventions to reduce falls should be undertaken. An example of such an integrated care pathway is provided in The Care of Patients with Fragility Fracture (‘Blue Book’), published by the British Orthopaedic Association and the British Geriatrics Society [132].

X-ray reports of vertebral fractures should be standardised to aid fracture identification.

FLS should include embedded local audit systems supported by a clinical fracture database to enable monitoring of care provided to fracture patients (e.g. Royal College of Physicians Fracture Liaison Services-Database (https://www.rcplondon.ac.uk/projects/fracture-liaison-service-database)).

FLS should be patient centred and integrated between primary and secondary care. Primary care physicians should follow-up patients at 4 and 12 months to review use of medications that increase the risk of falls and/or fracture, to ensure coprescription of calcium and vitamin D with bone-protective interventions and to monitor adherence to therapy [133]. FLS should include an educational intervention for patients and primary care physicians; however, education should not be the sole intervention (Evidence level 1a).

## Case finding and intervention thresholds

At present, there is no universally accepted policy for population-based screening to identify people with osteoporosis. With the recognition that factors in addition to BMD can improve fracture risk prediction, it is possible that screening strategies might be developed in the future and this is a recommendation for further research.

A trial of screening in the UK using FRAX (the SCOOP study), which has recently been completed but not yet reported in full, suggests promising effects of screening on treatment uptake and hip fracture risk [134, 135].

In the absence of a screening policy, a case-finding strategy is recommended where patients are identified because of a fragility fracture or by the presence of other clinical risk factors (Grade C recommendation). The use of risk factors that add information on fracture risk independently of BMD improves the predictive value of the assessment [30, 41] (Evidence level 1a).

Fracture risk should be assessed in postmenopausal women and men age 50 years or more with the risk factors outlined below where assessment would influence management (Grade C recommendation).

### Clinical risk factors considered in the FRAX assessment of fracture probability

- Age
- Sex
- Low body mass index (≤19 kg/m^2^)
- Previous fragility fracture, including morphometric vertebral fracture
- Parental history of hip fracture
- Current glucocorticoid treatment (any dose, by mouth for 3 months or more)
- Current smoking
- Alcohol intake 3 or more units daily
- Secondary causes of osteoporosis including:
  - Rheumatoid arthritis
  - Type I diabetes
  - Osteogenesis imperfecta in adults
  - Long-standing untreated hyperthyroidism
  - Hypogonadism/premature menopause (<45 years)
  - Chronic malnutrition
  - Chronic malabsorption
  - Chronic liver disease

Falls are an important risk factor for fracture but are not presently accommodated in the FRAX algorithm [136]. Additional common clinical risk factors that should alert to the possibility of high fracture risk are thoracic kyphosis and height loss (>4 cm) [137] (Evidence level 2) and type 2 diabetes [40] (Evidence level 1b). These, and other factors which have been associated with osteoporosis (either low BMD, fracture or both), and which may indicate the need for osteoporosis risk assessment outwith the FRAX algorithm, are listed in Table 5 [138].

**Table 5:** Risk factors for osteoporosis/ fractures not presently accommodated in FRAX

• Thoracic kyphosis
• Height loss (>4 cm)
• Type 2 diabetes
• Falls
• Inflammatory disease: ankylosing spondylitis, other inflammatory arthritides, connective tissue diseases
• Endocrine disease: hyperthyroidism, hyperparathyroidism, Cushing’s disease
• Haematological disorders/malignancy
• Muscle disease: myositis, myopathies and dystrophies
• Asthma, chronic obstructive pulmonary disease
• HIV infection
• Neurological/ psychiatric disease, e.g. Parkinson’s disease, multiple sclerosis, stroke, depression, dementia
• Nutritional deficiencies: calcium, vitamin D, magnesium, protein (note that vitamin D deficiency may contribute to fracture risk through undermineralisation of bone (osteomalacia) rather than osteoporosis)
• Medications
• Some immunosuppressants (calmodulin/calcineurine phosphatase inhibitors)
• (Excess) thyroid hormone treatment (levothyroxine and/or liothyronine). Patients with thyroid cancer with suppressed TSH are at particular risk
• Drugs affecting gonadal hormone production (aromatase inhibitors, androgen-deprivation therapy, medroxyprogesterone acetate, gonadotrophin hormone releasing agonists)
• Some antidiabetic drugs
• Some antipsychotics
• Some anticonvulsants
• Proton pump inhibitors

The approach recommended for decision making is based on fracture probabilities derived from FRAX and can be applied to men and women [139]. This approach is underpinned by cost-effectiveness analysis with generic alendronate as the intervention [140] (Evidence level 1b, Grade B recommendation). The assumptions used on cost-effectiveness are conservative and would permit the use of second line intervention in approximately 20% of patients.

**Fig. 2:**
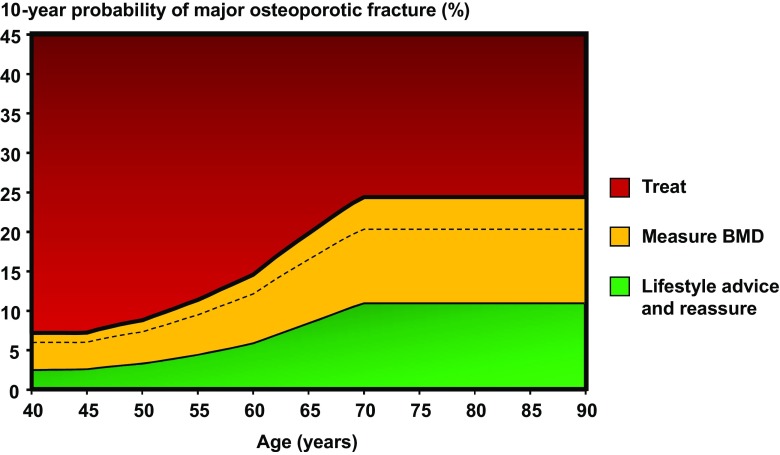
Graph showing assessment and intervention thresholds in the UK for major osteoporotic fracture probability. The *dotted line* represents the intervention threshold while the assessment thresholds are shown within the *amber area* [141]. *BPs* bisphosphonates, *GCs* glucocorticoids

Women with a prior fragility fracture should be considered for treatment without the need for further assessment, although BMD measurement is sometimes appropriate, particularly in younger postmenopausal women (Grade C recommendation). In men with or without a fragility fracture and in women without a previous fragility fracture, management strategy should be based on assessment of the 10-year probability of a major osteoporotic fracture (clinical spine, hip, forearm or humerus). Men and women with probabilities below the lower assessment threshold can be reassured. Men and women with probabilities above the upper assessment threshold can be considered for treatment. Men and women with probabilities between the upper and lower assessment threshold should be referred for BMD measurements and their fracture probability reassessed [4, 141]. The thresholds are shown in Fig. 2. In addition to the 10-year probability of a major osteoporotic fracture, the NOGG website also provides intervention thresholds that are based on the 10-year probability of hip fracture. Either or both thresholds can be used; indeed, the SCOOP study was based on treatment targeted on the basis of risk assessed by hip fracture probability [134].

The intervention threshold up to age 70 years is set at a risk equivalent to that associated with a prior fracture, in line with current clinical practice, and therefore rises with age. At age 70 years and above, fixed thresholds are applied [140, 141] (Grade B recommendation). The proportion of women potentially eligible for treatment rises from approximately 30 to 50% with age, largely driven by prior fracture prevalence [141] (Evidence level 1b). Fracture probabilities based on FRAX can be input into the website of the National Osteoporosis Guideline Group (www.shef.ac.uk/NOGG) to enhance management decisions.

The use of BMD assessments using this strategy makes more efficient use of resources than the scanning of all with risk factors [142] (Evidence level 1b). The strategy using the FRAX tool advantages more individuals at high risk and can be applied to men.

The Guideline Group is aware of the view that treatment should not be undertaken in women without recourse to a BMD test except in women with prior hip or vertebral fractures. The view arises because of a post hoc analysis showing reduced efficacy of alendronate in patients with BMD T-scores above −2.5 [143] (Evidence level 1b). However, other studies have shown little or no interaction of BMD on effectiveness of several agents, including some bisphosphonates, raloxifene and teriparatide [144, 145] (Evidence level Ib). Moreover, the clinical risk factors are not totally independent of BMD and, when clinical risk factors alone are used in women age 70 years or more to select patients at high risk, BMD is approximately 1 SD lower in the high-risk group compared with a low risk group [146] (Evidence level Ib). For several interventions (raloxifene and teriparatide), the response to treatment is independent of FRAX whereas in others (abaloparatide, bazedoxifene, denosumab, clodronate), the response is greater in patients with the higher fracture probabilities identified on the basis of clinical risk factors alone (Evidence level Ib).

Relatively simple arithmetic procedures are available which can be applied to conventional FRAX estimates of probabilities of hip fracture and a major fracture to adjust the probability assessment with knowledge of:

- High, moderate and low exposure to glucocorticoids [127] (Evidence level 2). See Table 3.
- Concurrent data on lumbar spine BMD [147] (Evidence level 1a). Increase/decrease fracture probability by 10% for each 1 standard deviation T-score difference between lumbar spine and total hip
- Information on trabecular bone score (TBS) [148] (Evidence level 1a). TBS values can be entered on the UK FRAX website.
- Hip axis length [149] (Evidence level 1b).
- Falls history [136] (Evidence level 2).

## Recommendations for training

It is recognised that osteoporosis is not subserved by any one specialty. The relevant specialties include rheumatology, orthopaedics, general practice, endocrinology, metabolic medicine, geriatrics and obstetrics and gynaecology. The problem is compounded by the fact that few specialties dealing with osteoporosis recognise training in osteoporosis and metabolic bone diseases as a component of higher professional training. It is recommended that this be given consideration by the relevant Royal Medical Colleges.

The issues associated with osteoporosis are also relevant to several specialties in nursing and other professions allied to medicine. It is recommended that the management of osteoporosis should be a component of training in all the relevant disciplines.

## Recommendations for commissioners of health care and the Department of Health

We recommend that commissioners of healthcare should recognise that fractures due to osteoporosis are a significant and growing public health issue, and ensure that they are dealt with explicitly in their local healthcare programme.

They should ensure that the local healthcare programme addresses approaches to reducing the prevalence of avoidable risk factors for osteoporosis and fractures related to falls and poor bone health and, in so doing, makes explicit the roles of both the NHS and other agencies.

They should ensure that accurate up-to-date information about the effects of pharmacological interventions is widely available to postmenopausal women and older men (≥50 years) and their professional advisers so that patients may make an informed decision about their use.

They should put arrangements in place so that those at particularly high risk of fragility fractures have the opportunity to receive appropriate investigation (e.g. fracture risk assessment, falls risk assessment, bone density measurement), life style advice (e.g. about diet, exercise and smoking) and bone-protective therapy.

They should bring together local specialists, generalists and other stakeholders, including patient representatives, to agree local treatment and referral practises for the management of osteoporosis and prevention of fragility fractures. It may be helpful to identify a lead clinician. The recommendations of the group should take account of local resources and relevant cost-effectiveness data. Guidelines should also be consistent with the evidence presented in this document. Once local guidelines have been agreed, they should be widely disseminated to relevant professionals and potential patients, and the necessary service changes made to allow the guidelines to be implemented. Implementation should be audited and appropriate changes in practice should be instituted where standards are not met.

As these guidelines will be adapted for local use, we recommend that criteria for monitoring compliance to the guidelines be developed.

We recommend that Clinical Commissioning Groups (CCGs) should specifically address the burden of fragility fractures on the local economy and ensure that Fracture Liaison Services are available for all patients sustaining a fragility fracture.

## Review criteria for audit

Documentation of the proportion of postmenopausal women and men age.

over 50 years presenting with risk factors for fragility fractures at primary care who receive formal fracture risk assessment.

Documentation of the proportion of postmenopausal women and men aged over 50 years with incident hip fracture who receive bone-protective medication within 6 months of fracture.

Participation in the Royal College of Physicians Fracture Liaison Service Database (https://www.rcplondon.ac.uk/projects/fracture-liaison-service-database). This is a national audit commissioned by the Healthcare Quality Improvement Partnership (HQIP) as part of the Falls and Fragility Fracture Audit Programme. The FLS-DB is included in the HQIP 2015/16 listing for national audits that must be reported both in the trust’s Quality Account and also form part of the National Clinical Audit Patient Outcomes Programme. All sites that treat fractures are eligible to participate.

## Summary of main recommendations

### Assessment of fracture risk

1. Fracture probability should be assessed in postmenopausal women, and men age 50 years or more, who have risk factors for fracture, using FRAX. In individuals at intermediate risk, bone mineral density (BMD) measurement should be performed using dual-energy X-ray absorptiometry and fracture probability re-estimated using FRAX.
2. Vertebral fracture assessment should be considered in postmenopausal women and men age > 50 years if there is a history of ≥4 cm height loss, kyphosis, recent or current long-term oral glucocorticoid therapy, or a BMD T-score ≤ −2.5.

### Lifestyle and dietary measures

1. A daily calcium intake of between 700 and 1200 mg should be advised, if possible achieved through dietary intake, with use of supplements if necessary.
2. In postmenopausal women and older men (≥50 years) at increased risk of fracture a daily dose of 800 IU cholecalciferol should be advised.
3. In postmenopausal women and older men receiving bone-protective therapy for osteoporosis, calcium supplementation should be given if the dietary intake is below 700 mg/day, and vitamin D supplementation considered in those at risk of or with evidence of vitamin D insufficiency.
4. Regular weight-bearing exercise should be advised, tailored according to the needs and abilities of the individual patient.
5. Falls history should be obtained in individuals at increased risk of fracture and further assessment and appropriate measures undertaken in those at risk.

### Pharmacological intervention in postmenopausal women

1. Alendronate or risedronate are first line treatments in the majority of cases. In women who are intolerant of oral bisphosphonates or in whom they are contraindicated, intravenous bisphosphonates or denosumab provide the most appropriate alternatives, with raloxifene or hormone replacement therapy as additional options. The high cost of teriparatide restricts its use to those at very high risk, particularly for vertebral fractures.
2. Treatment review should be performed after 3 years of zoledronic acid therapy and 5 years of oral bisphosphonate treatment. Continuation of bisphosphonate treatment beyond 3–5 years can generally be recommended in individuals age ≥75 years, those with a history of hip or vertebral fracture, those who sustain a fracture while on treatment, and those taking oral glucocorticoids.
3. If treatment is discontinued, fracture risk should be reassessed after a new fracture, regardless of when this occurs. If no new fracture occurs, assessment of fracture risk should be performed again after 18 months to 3 years.
4. There is no evidence to guide decisions beyond 10 years of treatment and management options in such patients should be considered on an individual basis.

### Glucocorticoid-induced osteoporosis

1. Women and men age ≥70 years, with a previous fragility fracture, or taking high doses of glucocorticoids (≥7.5 mg/day prednisolone) should be considered for bone-protective therapy.
2. In other individuals, fracture probability should be estimated using FRAX with adjustment for glucocorticoid dose.
3. Bone-protective treatment should be started at the onset of glucocorticoid therapy in individuals at high risk of fracture.
4. Alendronate and risedronate are first line treatment options. Where these are contraindicated or not tolerated, zoledronic acid or teriparatide are alternative options.
5. Bone-protective therapy may be appropriate in some premenopausal women and younger men, particularly in individuals with a previous history of fracture or receiving high doses of glucocorticoids.

### Osteoporosis in men

1. Alendronate and risedronate are first-line treatments in men. Where these are contraindicated or not tolerated, zoledronic acid or denosumab provide the most appropriate alternatives, with teriparatide as an additional option.
2. For estimation of fracture probability, femoral neck BMD T-scores in men should be based on the NHANES female reference database. When using the online version of FRAX for the estimation of fracture probability, femoral neck BMD values (g/cm^2^) should be entered and the manufacturer of the densitometer specified.

### Intervention thresholds for pharmacological intervention

1. The thresholds recommended for decision making are based on probabilities of major osteoporotic and hip fracture derived from FRAX and can be similarly applied to men and women.
2. Women with a prior fragility fracture can be considered for treatment without the need for further assessment, although BMD measurement may be appropriate, particularly in younger postmenopausal women.
3. Age-dependent intervention thresholds up to 70 years and fixed thresholds thereafter provide clinically appropriate and equitable access to treatment.

### Systems of care

1. Coordinator-based Fracture Liaison Services FLS should be used to systematically identify men and women with fragility fracture.

## Appendix 1

Guideline Development Writing Group:

The guideline development writing group was composed of two committees, the Guideline Development Group and the Expert Advisory Group. Members of both committees contributed to the content of the guideline, but voting on the recommendations was restricted to the Guideline Development Group. Disclosures of potential conflicts of interest of all members are available on the NOGG website (www.shef.ac.uk/NOGG).

No funding source/body was involved in the development of this guideline.

Guideline Development Group:

Juliet Compston (chair). Professor Emeritus of Bone Medicine, Cambridge Biomedical Campus, Cambridge UK

Alun Cooper: Primary Care Physician, Clinical Lead for Crawley Fracture Liaison Service, Crawley, Sussex

Celia Gregson: Consultant Senior Lecturer, Musculoskeletal Research Unit, University of Bristol & Honorary Consultant Geriatrician, Royal United Hospital NHS Foundation Trust, Bath, UK

Suzanne Hewitt: Patient representative (stepped down 3/2016)

David Reid: Emeritus Professor of Rheumatology, University of Aberdeen

Peter Selby: Endocrinologist, Consultant Physician and Honorary Clinical Professor of Metabolic Bone Disease, University of Manchester

Fizz Thompson, Clinical and Operations Manager, National Osteoporosis Society

Anne Thurston: Head of Policy, National Osteoporosis Society

Nic Vine: Public and patient representative

John Kanis: (ex officio) Professor Emeritus, Centre for Metabolic Diseases, University of Sheffield Medical School, Sheffield, United Kingdom and Professor, Institute for Health and Ageing, Catholic University of Australia, Melbourne, Australia

Expert Advisory Group:

Cyrus Cooper: Professor of Rheumatology, MRC Lifecourse Epidemiology Unit, University of Southampton and Professor of Musculoskeletal Science, University of Oxford

Neil Gittoes: Consultant and Honorary Professor of Endocrinology, University Hospitals Birmingham NHS Foundation Trust, Centre for Endocrinology, Diabetes and Metabolism, University of Birmingham and Birmingham Health Partners

Nicholas Harvey: Professor of Rheumatology and Clinical Epidemiology, and Honorary Consultant Rheumatologist, MRC Lifecourse Epidemiology Unit, University of Southampton

Sally Hope: Primary Care Physician, Clinical Assistant, Metabolic Bone, Nuffield Orthopaedic Hospital, Oxford

John Kanis: Professor Emeritus, Centre for Metabolic Diseases, University of Sheffield Medical School, Sheffield, United Kingdom and Professor, Institute for Health and Ageing, Catholic University of Australia, Melbourne, Australia

Eugene McCloskey: Professor in Adult Bone Disease and Honorary Consultant, University of Sheffield and Sheffield Director of the Centre for Integrated research in Musculoskeletal Ageing (CIMA), University of Sheffield

Kenneth Poole: Rheumatologist, Reader in Metabolic Bone Disease and Honorary Consultant Physician, Cambridge Biomedical Campus

## Appendix 2

List of stakeholders**:**

Arthritis Research UK

Association for Clinical Biochemistry and Laboratory Medicine

Bone Research Society

British Geriatrics Society

British Orthopaedic Association

British Orthopaedic Research Society

British Menopause Society

British Society for Rheumatology

European Calcified Tissues Society

International Osteoporosis Foundation

National Osteoporosis Society

Osteoporosis 2000

Osteoporosis Dorset

Primary Care Rheumatology Society

Royal College of General Practitioners

Royal College of Physicians

Royal Pharmaceutical Society

Society for Endocrinology

External reviewers**:**

Dr. Michael McClung, Associate Professor of Medicine, Oregon Osteoporosis Centre

Dr. William Leslie, Professor of Medicine and Radiology, University of Manitoba

Dr. Kassim Javaid, University Lecturer in metabolic Bone Disease, Nuffield Dept of Orthopaedics, Rheumatology and Musculoskeletal Sciences

Members of the Clinical and Scientific Committee of the National Osteoporosis Society

## Appendix 3

### Grading of recommendations

*Levels of evidence for studies of intervention are defined as follows:*

Ia from meta-analysis of randomised controlled trials (RCTs)

Ib from at least one RCT

IIa from at least one well-designed controlled study without randomisation

IIb from at least one other type of well-designed quasi-experimental study

III from well-designed non-experimental descriptive studies, e.g. comparative studies, correlation studies, case-control studies

IV from expert committee reports or opinions and/or clinical experience of authorities

*The validity of candidate risk factors is also assessed by an evidence-based approach:*

Ia Systematic reviews or meta-analysis of level I studies with a high degree of homogeneity

Ib Systematic reviews or meta-analysis with moderate or poor homogeneity

Ic Level I studies (with appropriate populations and internal controls)

IIa Systematic reviews or meta-analysis of level II studies

IIb Level II studies (inappropriate population or lacking an internal control)

IIIa Systematic reviews or meta-analysis of level III studies

IIIb Case-control studies

IV Evidence from expert committees without explicit critical scientific analysis or that based on physiology, basic research or first principles

*The quality of the guideline recommendations is similarly graded to indicate the levels of evidence on which they are based:*

Grade A evidence levels Ia and Ib

Grade B evidence levels IIa, IIb and III

Grade C evidence level IV

*Risk factors can also be categorised according to evidence for reversible risk:*

Grade A Validated by use as inclusion criteria in randomised controlled trials

Grade B Do not adversely affect fracture outcomes in randomised controlled trials

Grade C Untested or adversely affect intervention outcomes

## Appendix 4

**Table 6:** AMSTAR grading of systematic surveys and meta-analyses

Section	Reference	Type of study	AMSTAR rating
Fracture risk assessment	NICE CG 146 [29]	Systematic review	11/11
	Johansson et al. [42]	Meta-analysis	3/11
Lifestyle measures	Tai et al. [53]	Systematic review and meta-analysis	9/11
	Bolland et al. [55]	Systematic review	7/11
	Lewis et al. [57]	Meta-analysis	9/11
	Avenell et al. [60]	Systematic review	10/11
	Bischoff-Ferrari et al. [61]	Meta-analysis	9/11
	Bischoff-Ferrari et al. [62]	Meta-analysis	8/11
	Gillespie et al. [67]	Systematic review	10/11
	El-Khoury et al. [68]	Systematic review and meta-analysis	8/11
Pharmacological intervention	Crandall et al. [71]	Systematic review	9/11
Duration of therapy	Adler et al. [105]	Systematic review	4/11
	Khan et al. [115]	Systematic review	3/11
	Shane et al. [117]	Systematic review	5/11
Glucocorticoid-induced osteoporosis	Albaum et al. [120]	Systematic review	4/11
	Lekamwasam et al. [124]	Systematic review	7/11
	Amiche et al. [123]	Network meta-analysis	8/11
Fracture Liaison Services	Ganda et al. [130]	Systematic review and meta-analysis	5/11
Intervention thresholds	Kanis et al. [139]	Systematic review	8/11
